# Dyspnea as the First Manifestation of Silent Renal Cell Carcinoma

**DOI:** 10.30699/IJP.14.1.87

**Published:** 2018-12-27

**Authors:** Atieh Khorsand-Rahimzadeh, Fatemeh Khatami, Salma Sefidbakht, Hiva Saffar, Seyed Mohammad Tavangar

**Affiliations:** 1 *Dept. of Pathology, Shariati Hospital, Tehran University of Medical Sciences, Tehran, Iran*; 2 *Chronic Diseases Research Center, Endocrinology and Metabolism Population Sciences Institute, Tehran University of Medical Sciences, Tehran, Iran*

**Keywords:** Clear Cell Renal Cell Carcinoma, Neoplasm Metastasis, Pleura, Dyspnea

## Abstract

Renal cell carcinoma (RCC) accounts for only 3% of adult malignancies, and the lung is the most common site of metastasis of this tumor, which may be accompanied by pleural metastasis. However, solitary pleural involvement is very rare and its presentation with dyspnea as the first manifestation of RCC is extremely rare.

We describe a 39-year-old male with episodes of dyspnea dating back 6 months prior to hospital admission. During paraclinical investigations, chest computed tomography (CT) demonstrated pleural effusion and multiple pulmonary nodules, raising the question of primary mesothelioma or metastasis from distant focus. Histopathology and immuno- histochemical examinations of pleural biopsy provided evidence of metastatic RCC of the clear cell type. Therefore, an abdominal contrast computed tomography (CT) was performed, revealing a 3 cm right renal mass, which was then removed by partial nephrectomy.

Physicians and pathologists should be aware of unusual presentations of RCC with no symptoms attributable to the kidneys, including dyspnea as in our case.

## Introduction

RCC constitutes 3% of adult malignancies. Amongst various types of RCC, the clear cell type is the most common variant. It remains one of the greatest mimickers in pathology, and immunohistochemistry is a very helpful tool in its definite diagnosis (1). Approximately 30% of RCC patients present with metastatic diseases (2). Although the lung is the most common site of metastasis, dyspnea is rarely the initial clinical problem with this tumor (3).

Unlike the lung, the pleura is a rare site of metastasis for this tumor. In an autopsy-based study conducted by Saitoh, there was no solitary pleural involvement, whereas metastases exhibiting in both the lungs and pleura constituted 2% of cases with metastasis to two organs (4). Another study reviewed articles from 1967 to 2015, and found only 14 cases with solitary pleural involvement (5).

Being aware of this rare presentation of RCC and its mimickers will be a great help in achieving accurate e diagnoses, and as such, proper management of these patients. Here we report a case of clear cell RCC that presented with dyspnea as the first manifestation.

**Figure 1:**
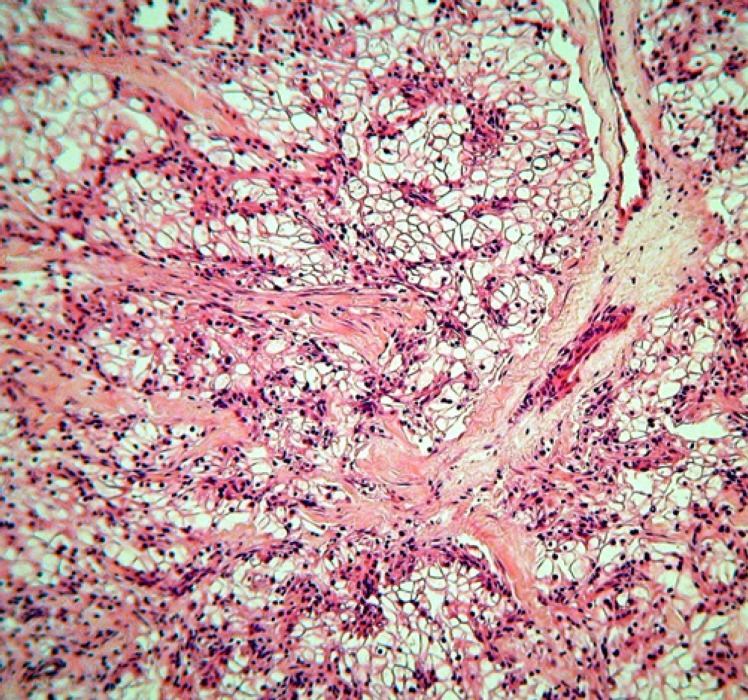
Pleural biopsy, hematoxylin and eosin staining, revealing neoplastic cells with clear cytoplasm (x100)

## Case Report

A 39-year-old man presented with episodes of dyspnea 6 months prior to hospital admission. His past medical and family histories were unremarkable.

Chest computed tomography (CT) revealed multiple pulmonary nodules in a diffuse pattern, accompanied by bilateral pleural effusion. The aspiration of the pleural effusion was bloody, and analysis of the fluid confirmed that it was an exudate.

A consulting pulmonologist suggested pleural biopsy. On gross examination, the specimen consisted of 4 ir- regular pieces of cream-gray, soft and rubbery tissue measuring 1.5x1x0.3 cm together.

On microscopic examination, sections from the pleural biopsy showed portions of neoplastic tissue, as well as fragments of fibrous connective tissue with mesothelium lining infiltrated by a neoplasm. The mentioned neo- plasm was composed of large cells with round to oval nuclei and a moderate amount of clear cytoplasm. Mild nuclear pleomorphism and intercellular capillary-sized vessels were two noteworthy findings (Figure.1).

**Figure 2:**
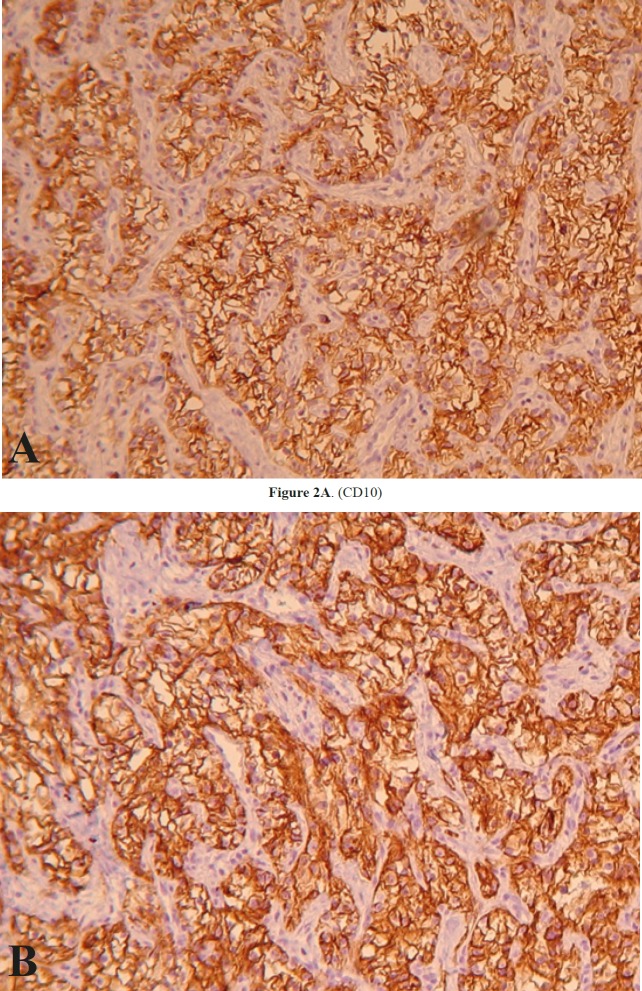
Immunohistochemistry of pleural biopsy (x100). A) CD10,diffusely positive in tumor cells. B) Carbonic anhydrase IX, diffusely positive in tumor cells

**Figure 3:**
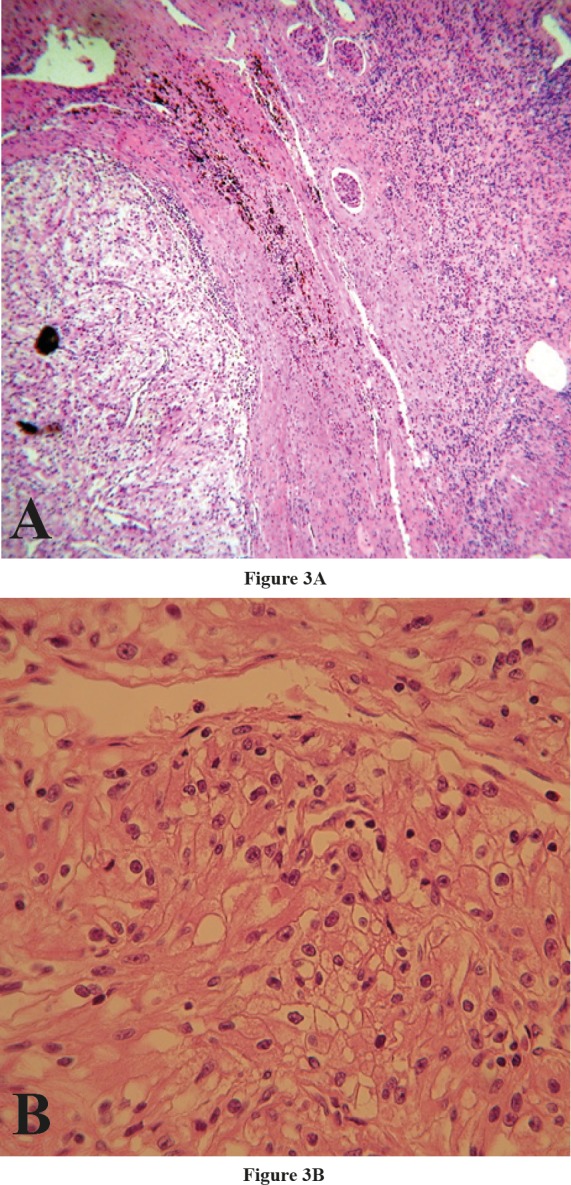
Nephrectomy specimen with hematoxylin and eosin staining, A) Low power view, revealing normal kidney and adjacent tumor (x50) B) High power view, demonstrating large cells with round to oval nuclei and clear cytoplasm (x400)

An immunohistochemistry study revealed the following results: tumor cells were strongly positive for CD10, Carbonic anhydrase IX, EMA, Pan CK and showed negative results for CK7, CK20, NapsinA, TTF1, Calretinin, WT1, CDX2, Inhibin and PSA (Figure.2).Based on histomorphologic findings and immunohistochemi- cal results, the tumor was diagnosed to be compatible with metastatic RCC, of the clear cell type. However we advised clinical/imaging correlation for the patient. Therefore, abdominal contrast computed tomography (CT) was performed, and showed a 3 cm right renal mass. Partial nephrectomy to remove the mass was performed. As suspected, the mass was of a clear cell RCC (Figure.3).

## Discussion

RCC accounts for 3% of adult malignancies (3). It is also called the “internist’s tumor” due to its variable pre- sentation and course. Beside the classic (but uncommon) triad of RCC presentation (hematuria, flank pain and flank mass), many rare first manifestations of this tumor have also been distinguished. Among these rare first clinical presentations are upper gastro-intestinal bleeding (6), obstructive jaundice (7), palpable warty urethral nodule (8), pleural effusion (9) and progressive dry cough (10). Likewise, our case presented with a rare mani- festation: dyspnea.

RCC can metastasize to almost any organ, including the lungs (most common), bones (11), brain (12), and in rare cases to the thyroid (13), adrenal gland (14), pituitary gland (15), and pleura(5, 16). Generally, as in our case which showed pleural involvement, solitary pleural lesions are rare and are often secondary to lung involvement (4, 5, 16), however another route of metastasis through Batson’s plexus has been reported (17, 18).

In a study by Saitoh on 1451 autopsy cases, in cases with single organ-metastasis, lung involvement was 32% and there was no solitary pleural involvement. However in the study’s total results, 154 (12%) cases had pleural metastasis (4).

In another study , Kataoka et al. showed that among 14 cases with solitary metastasis to the pleura, only one case revealed no pleural effusion and had long-term survival after resection (5). They also showed that cases with pleural effusion had a poor prognosis. Our case had pleural effusion combined with successful treatment. The latter might be due to the young age of our patient (39, in comparison to the mean age of 63 of cases, with the exception of one patient whose data was unavailable).

Histological examination and Immunohistochemical studies could be useful methods in diagnosing neoplastic and non-neoplastic disorders (19-21). Michael Daugherty et al. showed that the metastatic potential and size dependency depend on the histologic type. Among the most common histologic types, with increasing tumor size, clear cell is the most aggressive histology (22). Their study highlights the importance of accurate histologic typing.

In our case, the histomorphologic study on H&E sections revealed a clear cell neoplasm, which to some degree excluded the first impression of mesothelioma. In order to determine whether the tumor is primary or metastatic, and identify its origin, we performed IHC staining. Three groups of markers are recommended for a definite di- agnosis of renal cell carcinoma (some for detecting the site of origin, some for classification and some for prog- nostic use). In order to distinguish between primary and secondary lung tumors we performed CK7 and CK20 staining along with Napsin-A and TTF1. We also performed Calretinin and WT1 to exclude mesothelioma, CDX2 for the GI origin, Inhibin for the adrenal gland and PSA for the prostate. All the mentioned markers were negative in our case. In clear cell renal cell carcinoma, vimentin, AE1/AE3, CAM 5.2, and epithelial membrane antigen (EMA), and Carbonic anhydrase IX are positive and CK7, CD117 are negative (23). In our case, tumor cells were strongly positive for CD10, Carbonic anhydrase IX, EMA, and Pan CK, and the results revealed a diagnosis of clear cell RCC.

## Conclusion

In conclusion, RCC can initially present with several rare manifestations, and as in the reported case, pleural involvement is one of them. Although solitary pleural involvement during RCC is very rare, but as in our case, being aware of it may cause proper diagnosis and successful treatment.
